# Use of dental MRI for radiation-free guided dental implant planning: a prospective, in vivo study of accuracy and reliability

**DOI:** 10.1007/s00330-020-07262-1

**Published:** 2020-09-22

**Authors:** Tim Hilgenfeld, Alexander Juerchott, Johann M. E. Jende, Peter Rammelsberg, Sabine Heiland, Martin Bendszus, Franz S. Schwindling

**Affiliations:** 1 Department of Neuroradiology, Heidelberg University Hospital, Im Neuenheimer Feld 400, 69120 Heidelberg, Germany; 2 Department of Prosthodontics, Heidelberg University Hospital, Heidelberg, Germany

**Keywords:** Magnetic resonance imaging, Cone beam computed tomography, Dental implants, Humans, Tooth

## Abstract

**Objectives:**

To evaluate the accuracy and reliability of dental MRI for static guided implant surgery planning.

**Materials and methods:**

In this prospective study, a 0.4-mm isotropic, artifact-suppressed, 3T MRI protocol was used for implant planning and surgical guide production in participants in need of dental implants. Two dentists decided on treatment plan. Surgical guides were placed intraorally during a subsequent reference cone beam computed tomography (CBCT) scan. Inter-rater and inter-modality agreement were assessed by Cohen’s kappa. For each participant, dental MRI and CBCT datasets were co-registered to determine three-dimensional and angular deviations between planned and surgically guided implant positions.

**Results:**

Forty-five implants among 30 study participants were planned and evaluated (17 women, 13 men, mean age 56.9 ± 13.1 years). Inter-rater agreement (mean κ 0.814; range 0.704–0.927) and inter-modality agreement (mean κ 0.879; range 0.782–0.901) were both excellent for the dental MRI-based treatment plans. Mean three-dimensional deviations were 1.1 ± 0.7 (entry point) and 1.3 ± 0.7 mm (apex). Mean angular deviation was 2.4 ± 1.5°. CBCT-based adjustments of MRI plans were necessary for implant position in 29.5% and for implant axis in 6.8% of all implant sites. Changes were larger in the group with shortened dental arches compared with those for tooth gaps. Except for one implant site, all guides were suitable for clinical use.

**Conclusion:**

This feasibility study indicates that dental MRI is reliable and sufficiently accurate for surgical guide production. Nevertheless, more studies are needed to increase its accuracy before it can be used for implant planning outside clinical trials.

**Key Points:**

*• An excellent reliability for the dental MRI-based treatment plans as well as agreement between dental MRI-based and CBCT-based (reference standard) decisions were noted.*

*• Ideal implant position was not reached in all cases by dental MRI plans.*

*• For all but one implant site surgical guides derived from dental MRI were sufficiently accurate to perform implant placement (mean three-dimensional deviations were 1.1 ± 0.7 (entry point) and 1.3 ± 0.7 mm (apex); mean angular deviation was 2.4 ± 1.5°).*

## Introduction

Dental implants were introduced 50 years ago [1] and are now an established treatment option for the replacement of missing teeth [2]. In the USA, an adjusted increase in the prevalence of dental implants of 14% per year has been recorded, rising from 0.7% in 1999/2000 to 5.7% in 2015/2016 [3]. Dental implants can be planned by using either plain radiographs or cone beam computed tomography (CBCT) or computed tomography (CT). Prosthetically driven backward planning, also known as guided implant surgery, is currently of fundamental importance in implant surgery [4]. In this method, the three-dimensional (3D) position of the implant is determined by the optimum design of the future prosthetic restoration (e.g., the implant-supported single crown) and transferred into the patient by use of a surgical guide. To define the optimum prosthetically related implant position within the limits of available alveolar bone, 3D imaging is required [5, 6]. As the use of dental implants grows, the number of CBCT and CT examinations will also increase [7]. Compared with two-dimensional radiography like panoramic radiographs, however, the radiation dose of CBCT is nonetheless 2–200 times higher (range 10–1000 μSv; effective CBCT dose in this study, 211 μSv) [8, 9]. A meta-analysis identified the potential lifetime risks for thyroid cancer and meningioma posed by repeated X-ray-based imaging (two- and three-dimensional images) in dentistry [10]. In this context, dental MRI as a non-ionizing, cross-sectional imaging modality proofed to be a promising alternative for the two-dimensional evaluation/planning of implant sites as performed with panoramic radiographs, with a predefined implant position [11–13]. Previous studies concluded that measurement errors of dental MRI and CT are comparable for height and width measurements of jaw bones [13–16]. In prosthetically driven backward planning, however, the implant position is defined within the image dataset and isotropic imaging, as made available by CBCT, is necessary. The feasibility of dental MRI for backward planning has been show very recently in a case series [17]. The reliability and accuracy of dental MRI, however, has not yet been evaluated in a clinical setting. We wanted to test, therefore, whether implant planning decisions based on dental MRI would differ from those based on the reference imaging technique of CBCT and whether surgical guides derived from dental MRI would be sufficiently accurate to perform implant placement. The objectives of this study were therefore (I) to qualitatively evaluate the reliability and accuracy of dental MRI-based decisions regarding implant planning and (II) to quantitatively evaluate the accuracy of dental MRI-based surgical guides.

## Materials and methods

### Study sample

**Fig. 1:**
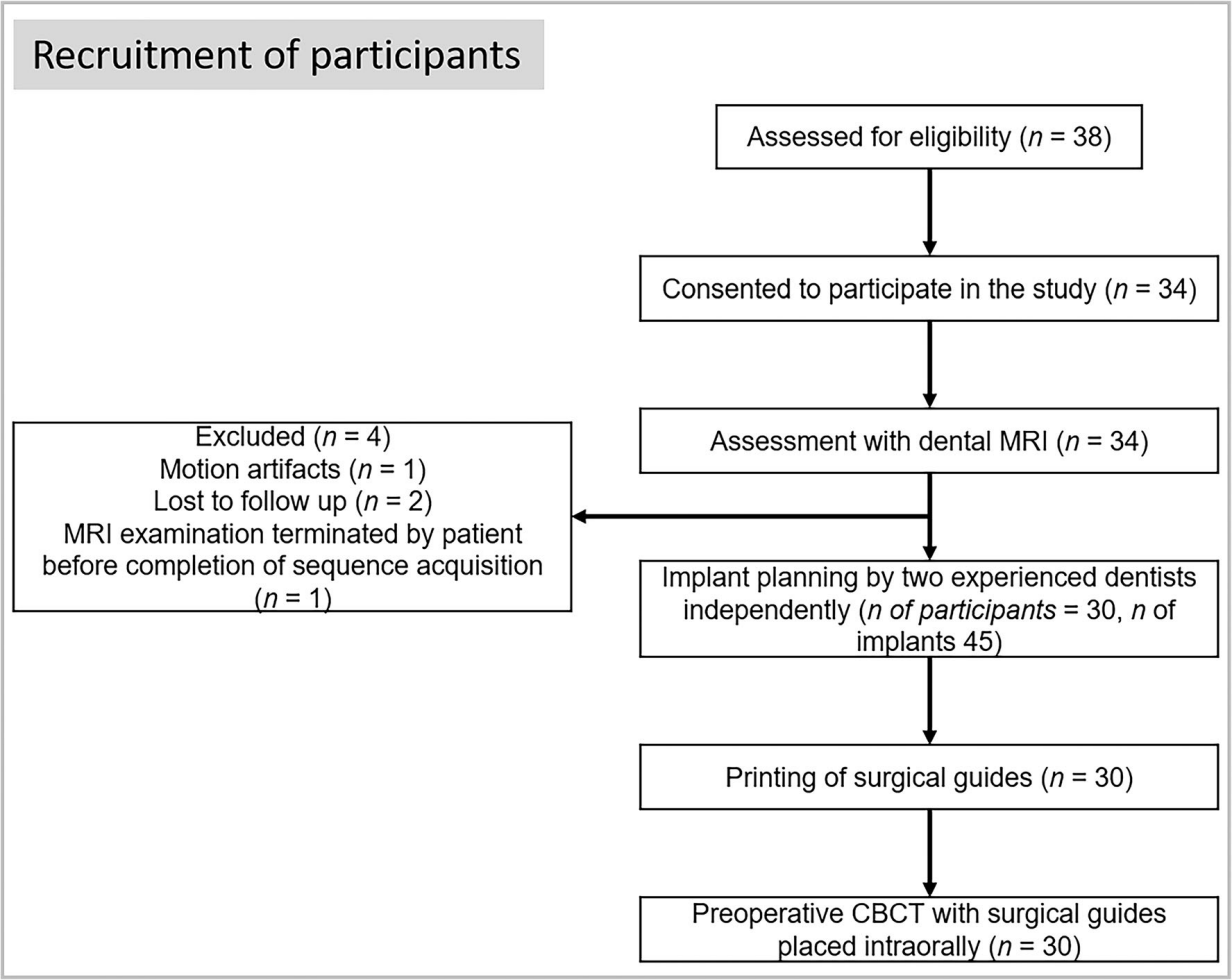
Flow chart illustrating the recruitment of participants

This prospective study was approved by the institutional ethics committee of Heidelberg University Hospital (approval number S-404/2014). Written informed consent was obtained from all participants. Potentially eligible participants were identified by means of a clinical examination, and 34 participants were consecutively enrolled in total (Fig. 1). The inclusion criteria were as follows: in need of a dental implant (including implant placement with simultaneous bone augmentation), teeth in both quadrants of the jaw (minimal number of three) to allow for reliable guide positioning, delayed implantation (at least 3 months after tooth extraction), and a stable medical condition to undergo implant surgery. The following criteria were grounds for exclusion: a two-stage surgical procedure with separate bone augmentation and implant insertion, contraindications to 3T MRI, age below 18 years, pregnancy, and claustrophobia. The study was planned and performed according to the STARD guidelines [18].

### Planning procedure for implant placement

**Fig. 2:**
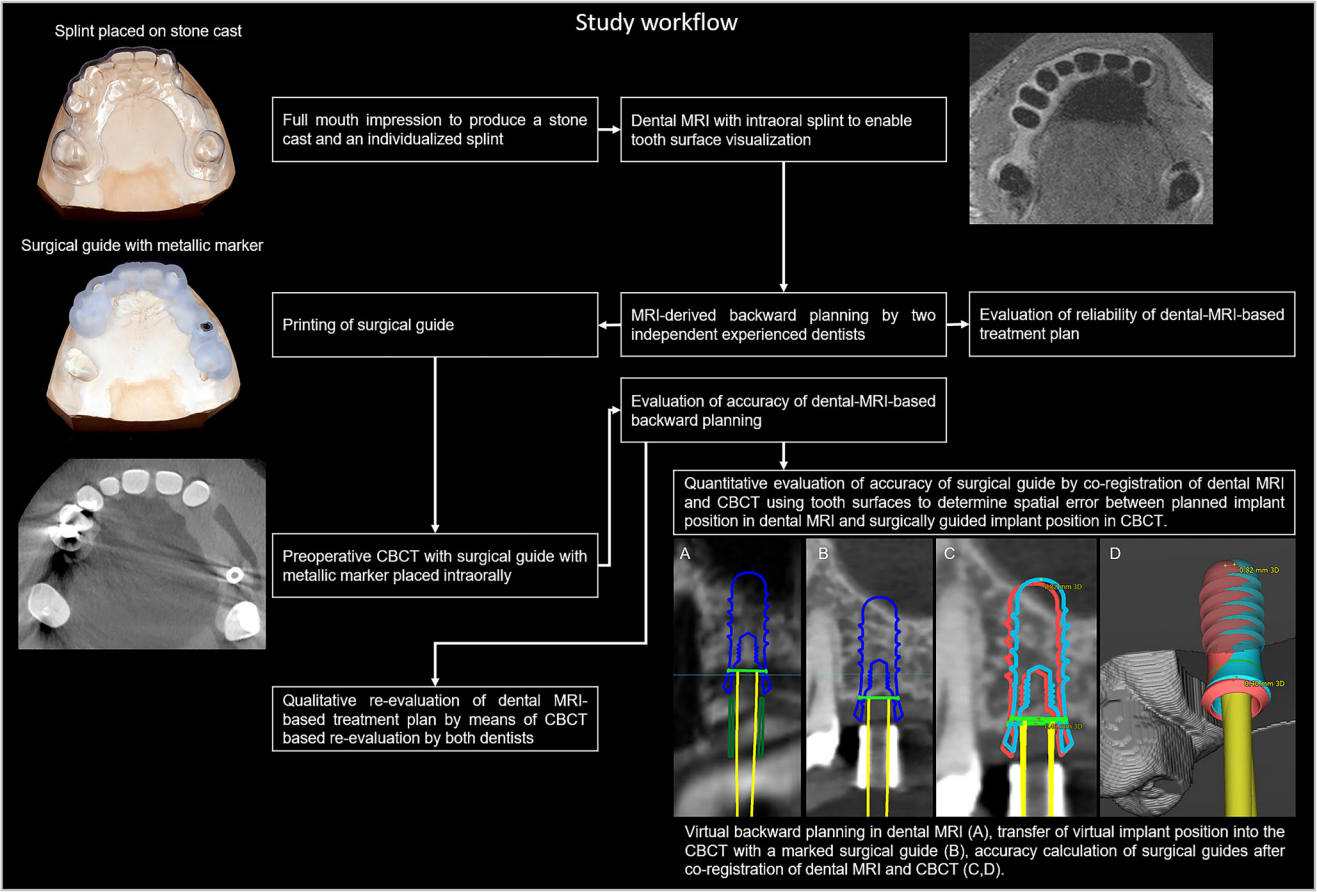
Flow chart illustrating the workflow of the study

Full-arch impressions (Impregum, 3M GmbH) to obtain a stone cast and a 0.4-mm isotropic, artifact-suppressed, proton-weighted dental MRI scan of the relevant jaw were taken for each participant (Fig. 2). To enable accurate segmentation of tooth surfaces in MRI data, a splint technique was used during the dental MRI examination, as described previously [19]. DICOM data from the dental MRI and STL data from the digitalized stone cast (D2000, 3shape) were subsequently imported and co-registered in an established software program for guided implant surgery (coDiagnostiX 9.12, Dental Wings Inc.). To assess the intra-modality reliability, backward planning was performed independently by two experienced dentists with more than 7 and 20 years’ experience of implant planning, respectively. Differences between decisions were clarified in a consensus reading. The dentists were asked to determine a treatment plan (implant type and dimensions, need for and type of bone augmentation, and implant position and axis) in accordance with manufacturer specification of the implants and established clinical criteria [20, 21]. If bone augmentation was required, the type of augmentation had to be specified (spread, split, bone chips, block, sinus lift, condensing, or any combination of the aforementioned procedures). After selecting the optimum implant position and axis, a tooth-supported guide with a thickness of 3 mm was designed, exported as STL, and 3D printed for each participant (Pro2, Asiga). Finally, each participant underwent a CBCT scan for the purposes of guided implant planning.

### Image acquisition parameters

Dental MRI examinations were performed with a 3T MRI system (Magnetom Tim-Trio, Siemens Healthcare) using a dedicated 15-channel dental coil (Mandibula, Noras MRI products GmbH). A multi-slab acquisition with view-angle tilting gradient was used, based on a sampling perfection with application-optimized contrasts using different flip-angle evolution (MSVAT-SPACE) prototype sequence (repetition time 1170 ms, echo time 6.4 ms; field of view 168 × 131 mm^2^; voxel size 0.4 × 0.4 × 0.4 mm^3^; matrix 384 × 300; slice oversampling 220%; slices 80; acquisition time 7.45 min). This MRI technique uses slab-selective excitation and refocusing radiofrequency pulses that enable interleaved multi-slab acquisition [22]. The technique was specifically optimized and evaluated for high spatial resolution, artifact-suppressed dental MRI, as described previously [23].

CBCT imaging (3D Accuitomo 170, J Morita) was performed as follows: field of view 8 × 8 cm^2^; tube voltage 90 kV; tube current 7 mA; 14 bit; 360° rotation in 17 s; 560 frames; and an isotropic voxel size of 160 μm.

### Accuracy evaluation

To transfer the MRI-based implant positions and axes into the CBCT datasets, surgical guides with a metal marker were placed intraorally during the CBCT scans (Fig. 2). Wooden spatulas were additionally placed intraorally to ensure a stable support of all opposite teeth on the surgical guide. The qualitative accuracy was assessed by re-evaluation of MRI-derived implant position and the MRI-derived treatment plan in CBCT by both dentists together, according to the same criteria they were using for the MRI-based planning. For the quantitative accuracy analysis, the CBCT data were imported into the same implant planning software and co-registered with the dental MRI using the tooth surfaces as references. The marker within the surgical guide on the CBCT examinations was used to identify the surgically guided implant position. Afterwards, the planned implant position in the dental MRI datasets was compared with the surgically guided implant position in the CBCT images (Fig. 2). Finally, the 3D deviation of the entry point and implant apex were calculated, as was the deviation of the implant axis.

### Statistical analysis

Because this is a prospective feasibility study, no sample size calculation was possible, and *p* values are descriptive in nature. To determine inter-rater and inter-modality reliability, Cohen’s kappa (κ) and the 95% confidence interval (CI) were used and interpreted as described previously [24]. Data were analyzed using SPSS (version 25, 2017, SPSS Inc.). Differences in accuracy between implant sites used to restore shortened dental arches and those in tooth gaps were analyzed using Mann-Whitney *U* or two-tailed Student *t* test, depending on data distribution in Shapiro-Wilk test. A level of significance was defined at *p* ≤ 0.05.

## Results

### Participant characteristics

In total, 34 participants were consecutively enrolled in this study. Four participants could not be included in the final analysis (Fig. 1). Thus, 45 implant sites among 30 participants were planned and evaluated in total (17 women, 13 men, mean age 56.9 ± 13.1 years). Implants were placed between neighboring teeth in 26 sites and used to restore shortened dental arches in 19 sites (i.e., implant splints were supported distally by soft tissue only).

### Planning accuracy of dental MRI

**Fig. 3:**
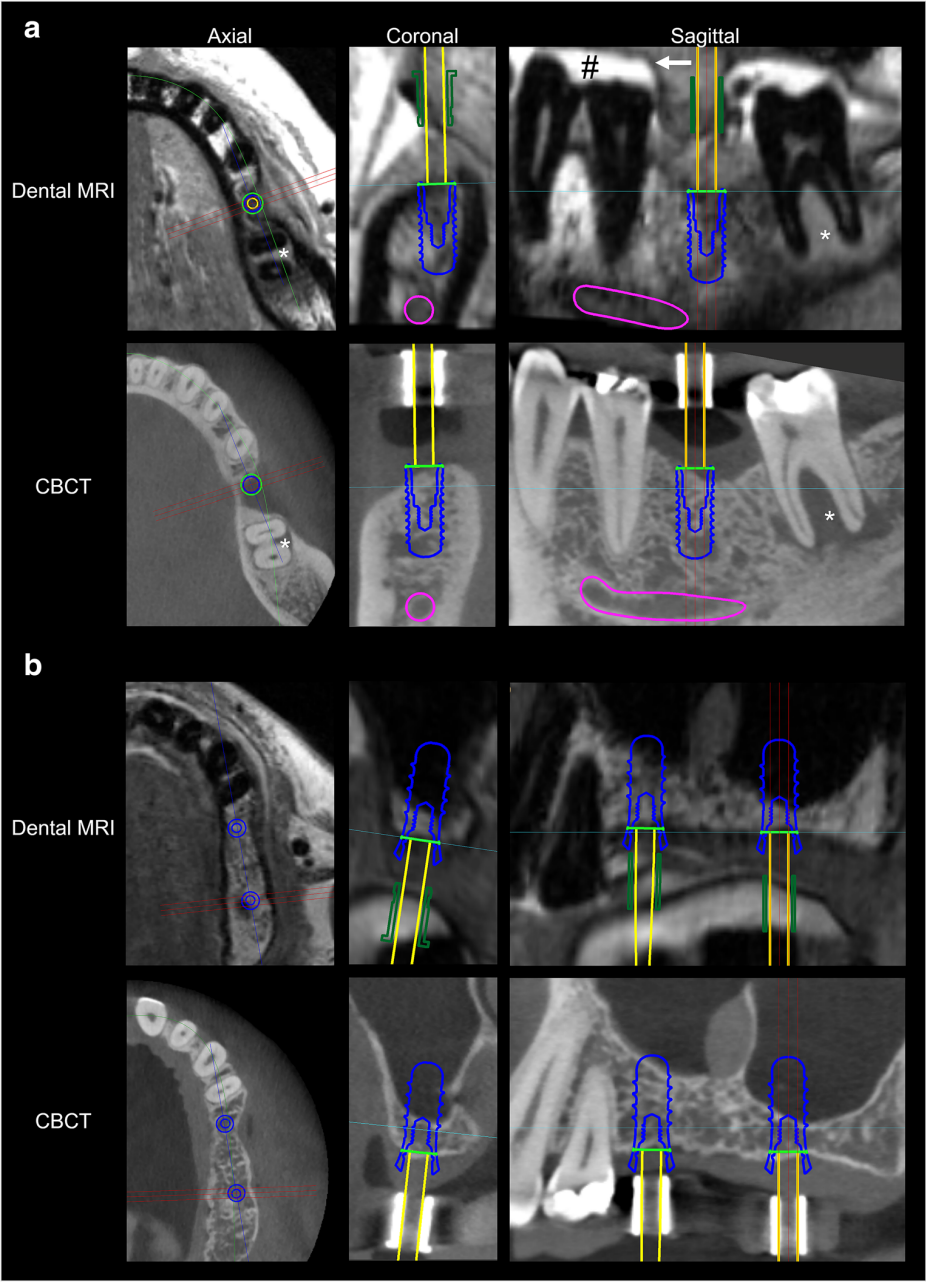
Direct comparison of two examples of implant planning in dental MRI and CBCT. In example **a**, implant insertion was planned in region 36. Based on dental MRI and CBCT images, no bone augmentation was necessary. In addition, a large combined periodontal-endodontic lesion can be seen in region 37 in both imaging modalities. Note the good delineation of the tooth surfaces against the bright toothpaste (#) in the splint (arrow). In example **b**, implant insertion was planned in regions 25 and 27 (coronal images from region 27). For both imaging modalities, it was decided that sinus lift augmentation was necessary in both regions

**Fig. 4:**
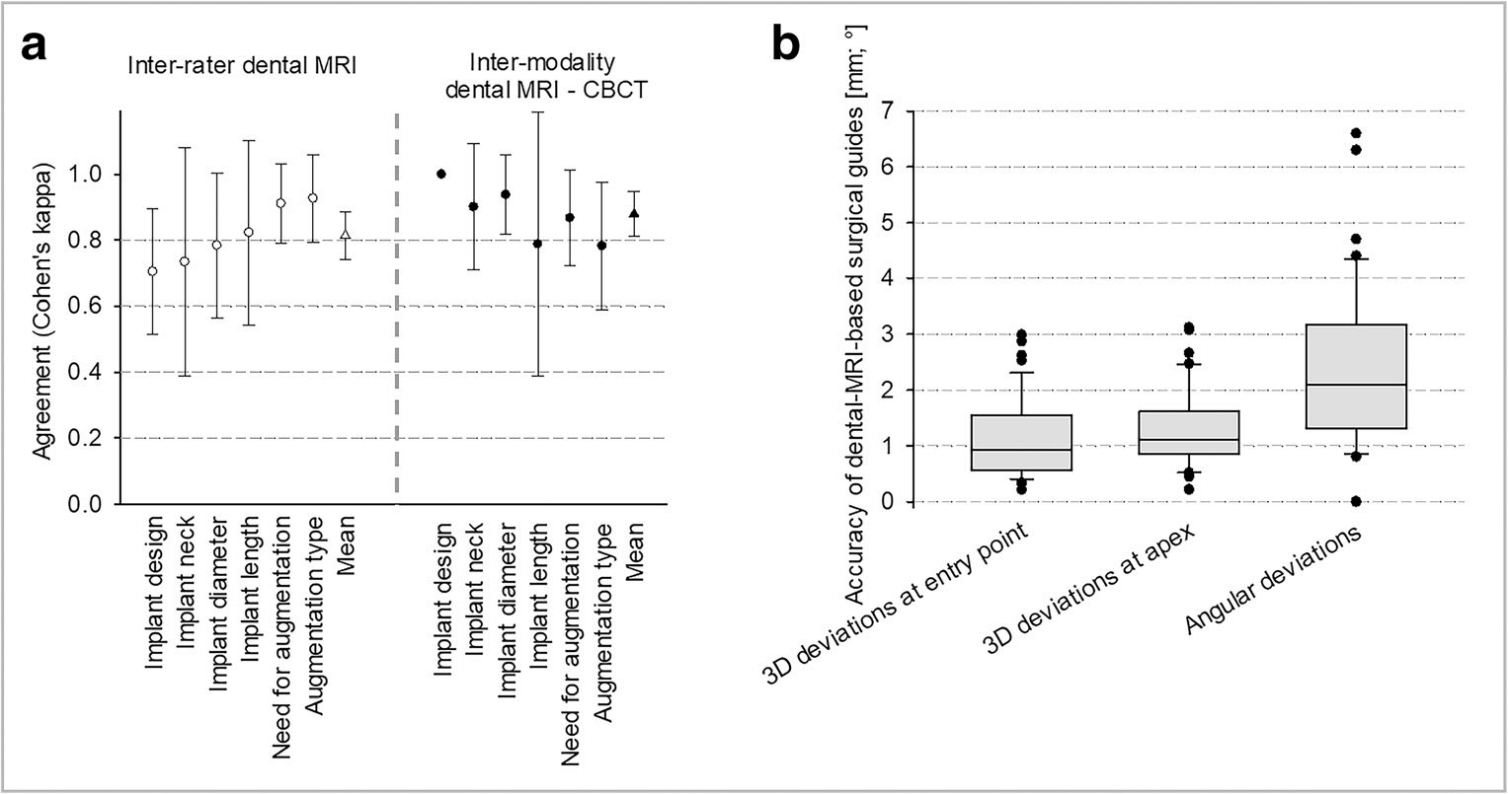
Inter-rater reliability of dental MRI-based treatment plan and inter-modality accuracy of dental MRI-based decisions compared with CBCT (**a**). Accuracy of dental MRI-based surgical guides (**b**)

**Fig. 5:**
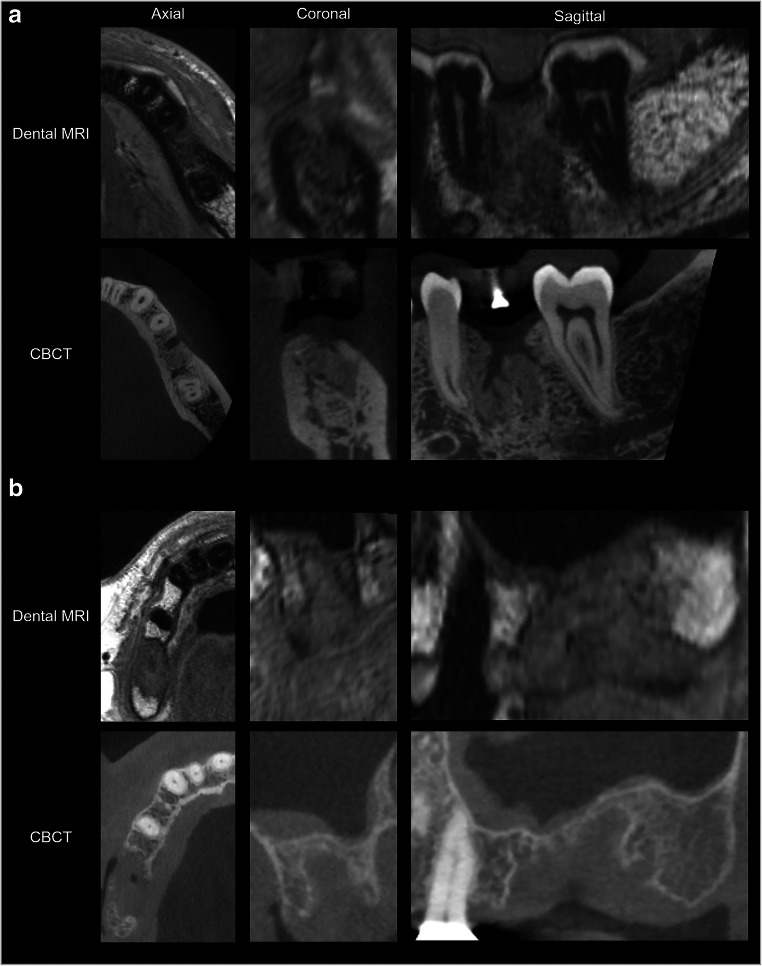
Two cases of extensive, bone loss in dental MRI and CBCT. Based on dental MRI, case **a** was misclassified as sufficient bone without the need for bone augmentation. At implant site 36, the preserved outer shape of the alveolar bone was misleading. In the second case (**b**), dentists correctly identified extensive bone loss in implant site 17 from the dental MRI examination. It was confirmed by CBCT that simultaneous implant insertion and bone augmentation were therefore not possible

The high spatial resolution protocol produced detailed images of the jaws that could be assessed in all three dimensions without a reduction in image quality (Fig. 3). Consequently, mean inter-rater agreement (mean κ 0.814; 95% CI 0.741–0.887; Fig. 4a) and mean inter-modality agreement between dental MRI-based and CBCT-based decisions (mean κ 0.879; 95% CI 0.811–0.948) were both excellent. After re-evaluation of the dental MRI-based plans by means of comparison with CBCT, no changes were made with regard to implant type, neck type, implant length, or implant diameter for 43 out of 45 planned implant sites (inter-modality κ range 0.788–1; Fig. 4a). For one site, the implant diameter and neck design were changed (from 4.1 to 4.8 mm and from a regular neck to a wide neck, respectively). With regard to another site, an initial implant length of 8 mm was changed to 10 mm. Bone augmentation procedures were performed for more than 50% of implant sites (23 out of 45; Table 1). For 42 of the 45 implant sites, the decision whether to perform bone augmentation or not was made correctly on the basis of dental MRI planning (κ 0.867; 95% CI 0.722–1). For three sites, the need for bone augmentation was not predicted by dental MRI, but was subsequently identified based on CBCT images (negative predictive value (NPV) 0.88; see example in Fig. 5a). For one implant site, extensive bone loss was predicted by dental MRI correctly. Consequently, the implant site was classified as unsuitable for simultaneous bone augmentation and implant placement (Fig. 5b). Dental MRI-based planning did not incorrectly predict the need for bone augmentation for any site (based on re-evaluation after CBCT; positive predictive value (PPV) 1). For all implant sites where the need for bone augmentation was identified on the basis of dental MRI (*n* = 20), the decision was confirmed by CBCT (20 out of 20 sites; κ for all 23 augmented sites = 0.782; 95% CI 0.588–0.976).

**Table 1:** Overview of all planned implant sites and their respective accuracy of surgically guided implant position

Patient	Implant site		Kennedy class	Bone augmentation		Corrections after CBCT		Accuracy of MRI-derived surgical guide		
Number	Neighboring teeth	Position		Based on MRI	Based on CBCT	Position of entry point [mm]	Angulation [°]	3D deviation at entry point [mm]	3D deviation at apex [mm]	Angular deviation [°]
1	Free-ending	15	1	Sinus lift	Sinus lift	2.0	10	1.3	1.3	1.6
	Free-ending	26	1	Sinus lift	Sinus lift	1.0	–	1.2	1.4	2.2
2	Free-ending	36	1	No	No	–	–	1.8	2.1	3.8
	Free-ending	46	1	No	No	–	–	0.4	1.2	4.7
3	Free-ending	45	2	No	No	1.0	–	1.3	1.9	3.7
	Free-ending	47	2	No	No	–	–	2.1	3.1	6.6
4	Free-ending	14	2	No	No	8.0	–	0.9	1.0	1.3
	Free-ending	16	2	Sinus lift	Sinus lift	–	–	0.3	0.5	1.1
5	Free-ending	26	2	Sinus lift	Sinus lift	1.0	–	1.8	2.0	1.3
6	Free-ending	35	2	No	No	–	–	0.2	0.2	0.0
	Free-ending	37	2	Bone chips	Bone chips	–	–	0.5	0.7	1.4
7	Free-ending	25	2	Sinus lift	Sinus lift	–	–	0.4	0.8	3.2
	Free-ending	27	2	Sinus lift	Sinus lift	–	–	0.3	0.5	1.7
8	Free-ending	37	2	No	Bone condensing	1.0	10	0.8	1.1	2.0
9	Free-ending	44	2	No	No	2.0		0.7	0.9	2.5
	Free-ending	47	2	No	No	1.0	10	1.3	1.6	2.5
10	Free-ending	37	2	No	No	–	–	2.9	3.1	6.3
11	Free-ending	46	2	Bone split	Bone split	1.5	–	1.2	1.6	2.8
Mean ± standard deviation [mm]						1 ± 1.9	1.7 ± 3.8	1.1 ± 0.7	1.4 ± 0.8	2.7 ± 1.8
3	Tooth gap	36	2	No	No	–	–	1.1	1.3	2.9
11	Tooth gap	35	2	No	No	–	–	1.2	1.6	3.1
	Tooth gap	37	2	No	No	–	–	1.7	2.1	3.7
12	Tooth gap	17	3	No	No	–	–	0.6	0.8	1.5
	Tooth gap	25	3	No	No	–	–	0.9	1.1	1.2
13	Tooth gap	45	3	Bone split	Bone split	–	–	2.6	2.5	1.8
	Tooth gap	47	3	Bone split	Bone split	2.0	–	3.0	2.5	4.3
14	Tooth gap	26	3	Sinus lift	Sinus lift	–	–	0.6	1.0	3.0
15	Tooth gap	36	3	No	No	–	–	1.0	1.1	0.8
16	Tooth gap	46	3	Bone split	Bone split	–	–	1.0	1.1	0.9
	Tooth gap	47	3	Bone split	Bone split	–	–	1.1	1.1	0.0
17	Tooth gap	15	3	No	No	–	–	2.0	1.6	3.8
18	Tooth gap	21	3	No	No	–	–	0.4	0.4	0.0
19	Tooth gap	45	3	No	No	–	–	0.7	0.8	1.1
20	Tooth gap	36	3	No	Split and chips	1.0	–	0.6	0.7	1.4
21	Tooth gap	25	3	Sinus lift	Sinus lift	–	–	1.6	1.6	1.0
	Tooth gap	26	3	Sinus lift	Sinus lift	–	–	1.7	1.6	1.6
22	Tooth gap	47	3	Bone split	Bone split	1.0	–	0.5	0.5	2.5
23	Tooth gap	14	3	Sinus lift	Sinus lift	–	–	0.5	1.3	4.4
24	Tooth gap	47	3	No	No	–	–	0.9	1.1	1.9
25	Tooth gap	26	3	Sinus lift	Sinus lift	–	–	0.9	1.0	1.4
26	Tooth gap	36	3	No	No	1.0	–	2.5	2.7	3.1
27	Tooth gap	26	3	Sinus lift	Sinus lift	–	–	0.6	0.9	2.7
28	Tooth gap	36	3	No	No	–	–	0.6	1.0	2.4
29	Tooth gap	46	3	No	Bone split	–	–	0.8	1.1	3.5
30	Tooth gap	26	3	Sinus lift	Sinus lift	–	–	0.8	0.8	1.2
Mean ± standard deviation [mm]						0.2 ± 0.5	0 ± 0	1.1 ± 0.7	1.3 ± 0.6	2.1 ± 1.3
*p* value (free-ending vs. tooth gap)						0.012^a^	0.035^a^	0.659^a^	0.821^a^	0.207^b^

^a^Mann-Whitney *U* test

^b^Two tailed Student *t* test

For 31 out of 44 sites (70.5%), the planned implant position was not altered after the CBCT-based re-evaluation. Small changes were made to the implant position of 13 sites (29.5%; mean change of 1.8 mm) and to the implant axis of three sites (6.8%; mean change of 10°). The subgroup analysis of implants placed in tooth gaps versus implants placed to restore shortened dental arches revealed significant differences (Table 1). Significantly less corrections were performed by both dentists for the implants placed in tooth gaps compared with implants placed in free-ending positions for both, the CBCT-based corrections of the entry point (0.2 ± 0.5 vs. 1 ± 1.9 mm; *p* = 0.01) and angulation (0 ± 0° vs. 1.7 ± 3.8; *p* = 0.04). If the single 8 mm outlier in the free-end subgroup (in dental MRI-missed potential remainder of a tooth root measuring 1 mm in CBCT), however, is excluded, the accuracy of that subgroup (0.6 ± 1.9 mm) is close the tooth gap subgroup and the mean change of all implants is reduced from 1.8 to 1.3 mm. As all changes, except one, affected the second (unguided) drill, and not the first (guided) drill, 97.8% of guides (43 out of 44) were classified as suitable for implant surgery after qualitative evaluation of dental MRI.

Evaluation of the quantitative accuracy of the dental MRI-based surgical guides for the 44 planned implants revealed mean 3D deviations of 1.1 ± 0.7 mm at the entry point (minimum 0.2 mm; maximum 3 mm) and 1.3 ± 0.7 mm at the implant apex (minimum 0.2 mm; maximum 3.1 mm; Fig. 4b). In addition, a mean angular deviation of 2.4 ± 1.5° (minimum 0°; maximum 6.6°) was observed. Slightly larger deviations were found for implant sites with shortened dental arches vs. implant sites in tooth gaps for the 3D deviation of entry point/implant apex, as well as the angulation, without reaching statistical significance (Table 1).

## Discussion

This study shows that dental MRI-based backward planning is highly reliable and results in sufficiently accurate surgical guides. It also showed, however, that the method is not yet capable to achieve the highest prosthetic and surgical demands on treatment planning in all cases.

This study has several methodological strengths. First, it made use of a previously established technique to visualize tooth surfaces within dental MRI [19]. As a result, it was possible to integrate dental MRI into an existing digital workflow without the need for additional software or time-consuming postprocessing steps. Second, it used a single-scan protocol with an examination time of less than 10 min. This has the potential to reduce examination costs. Moreover, in contrast to the earlier studies from Gray et al and Pompa et al studies [11, 13], we voted for a dedicated dental coil instead of a standard head and neck coil. The higher signal-to-noise ratio [25, 26] allowed for smaller and isotropic voxel size (440 μm isotropic). The latter is a prerequisite for backward planning, as multiplanar reconstructions are essential for finding the correct implant axis in relation to available bone and prosthodontics demands at the same time. The chosen sequence differed slightly from Flügge et al who used a SPACE and not a MSVAT-SPACE sequence. The MSVAT-SPACE sequence, however, offered the advantage of 56% less susceptibility artifacts compared with the SPACE in a previous study [23]. This likely results in improved tooth surface reconstructions directly adjacent to metallic crowns, pontics, or implants which are frequently observed in patients in need of dental implants. Thereby, it contributes to increase the accuracy and applicability of dental MRI-based backward planning. Finally, the accuracy of the dental MRI-based treatment plans was directly compared with the clinical reference imaging modality of CBCT.

Although dental MRI-derived implant planning resulted in accurate decisions regarding implant type, dimensions, and type of bone augmentation for most implant sites, it must be noted that the planned implant position and angle were changed slightly (for 29.5% and 6.8% of implant sites, respectively) at the stage of unguiding drilling after CBCT re-evaluation. In addition, three participants in need of bone augmentation were not identified as such from dental MRI images (NPV 0.88; PPV 1). Without the CBCT for re-evaluation, this would have resulted in a moderate extension of operation time in two cases and in a failed implantation in one case. This might be because partially calcified tissues appear different in CBCT images than they do in MRI images, especially if cortical bone borders are still intact as in our case. Consequently, the volume of bone available can be overestimated from dental MRI images. Implant planning based on dental MRI might therefore require the involvement of dentists and oral surgeons with sufficient experience of interpreting dental MRI images or a learning curve has to be acknowledged, respectively. Moreover, other sequence techniques may offer improved evaluation of bone, like ultrashort or zero time of echo sequences [27]. However, other disadvantages may come along with such sequences like lower resolution, lower image quality, and more susceptibility artifacts compared with MSVAT-SPACE [23].

The subgroup analysis revealed more and significantly larger corrections of MRI-derived implant position/angulation in free-ending positions compared with implant sites between neighboring teeth and slightly less accurate MRI-derived surgical guides (not statistically significant). As the spatial distribution of available teeth surfaces for co-registration is limited in patients with shortened arches, the co-registration of MRI and digitalized impression data might be less accurate in these patients, leading to a less precise transfer of the virtual implant position into the surgical guide. That result is in accordance with previous studies which reported a similar dependency of the accuracy and the number of residual teeth [28, 29].

The authors are not aware of any similar studies that have evaluated the accuracy of CBCT-based templates in vivo, most likely because of ethical concerns associated with a second preoperative CBCT scan. However, one ex vivo study by Kühl et al on CBCT-derived template accuracy is available for comparison [30]. Their study investigated the accuracy of surgical guides printed from cast models using the same planning and evaluation software ex vivo. For a 10-mm implant (the most frequently used implant length in our study), Kühl et al reported an apical deviation of 0.49 mm (minimum 0.13, maximum 1.09 mm). By comparison, apical deviation in our study was larger (mean 1.3 ± 0.7; minimum 0.2; maximum 3.1 mm). This may be due to our in vivo setting (i.e., incorporating motion artifacts) and a lower scanning resolution (0.4 mm isotropic; accuracy of optical scanner used by Kühl et al: approximately 15 μm). Another source of errors in our study was registration accuracy, which had an effect twice: once in the implant planning procedure (registration of dental MRI data with digitalized tooth models) and once in the quantitative assessment of surgical guide accuracy (registration of dental MRI with CBCT). For registration in implant planning, tooth surfaces are commonly used. The errors for in vivo tooth surface reconstructions derived from dental MRI and CBCT (mean error ± root mean square of dental MRI and CBCT 0.26 ± 0.1 and 0.1 ± 0.04 mm, respectively) were reported in a recent study [19]. This explains, at least in part, why accuracy was lower in our study than in Kühl et al.

Several limitations of this particular application of dental MRI must be addressed. The value of our reliability assessment is limited to some extent, as both surgeons were working in the same department. Moreover, the costs of dental MRI currently restrict its clinical use.

In conclusion, our feasibility study contributes to the current literature by providing evidence that dental MRI-based backward planning is reliable and results in surgical guides sufficiently accurate for implant placement. More research, however, is necessary to increase the accuracy of dental MRI, for example, by increasing spatial resolution or decreasing acquisition time to reduce motion artifacts. These findings may help to facilitate prosthetically driven backward implant planning without radiation exposure. This is particularly important in relation to younger individuals, who are more sensitive to radiation. However, more studies regarding dental MRI and implant placement are required before this imaging modality can be used outside clinical studies.

## Abbreviations

CBCT: Cone beam computed tomography
CI: Confidence interval
κ: Cohen’s kappa
